# Risk Factors Promoting External Ventricular Drain Infections in Adult Neurosurgical Patients at the Intensive Care Unit—A Retrospective Study

**DOI:** 10.3389/fneur.2021.734156

**Published:** 2021-11-08

**Authors:** Farjad Khalaveh, Nadia Fazel, Mario Mischkulnig, Matthias Gerhard Vossen, Andrea Reinprecht, Christian Dorfer, Karl Roessler, Johannes Herta

**Affiliations:** ^1^Department of Neurosurgery, Medical University of Vienna, Vienna, Austria; ^2^Department of Medicine I, Division of Infectious Diseases and Tropical Medicine, Medical University of Vienna, Vienna, Austria

**Keywords:** external ventricular drain, EVD associated infection, colonization, contamination, risk factors promoting EVD infections, ventriculitis

## Abstract

**Objectives:** Multiple risk factors have been described to be related to external ventricular drain (EVD) associated infections, with results varying between studies. Former studies were limited by a non-uniform definition of EVD associated infection, thus complicating a comparison between studies. In this regard, we assessed risk factors promoting EVD associated infections and propose a modified practice-oriented definition of EVD associated infections.

**Methods:** We performed a retrospective, single-center study on patients who were treated with an EVD, at the neurosurgical intensive care unit (ICU) at a tertiary center between 2008 and 2019. Based on microbiological findings and laboratory results, patients were assigned into an infection and a non-infection group. Patient characteristics and potential risk factors were compared between the two groups (*p* < 0.05). Receiver operating characteristics (ROC) for significant clinical, serum laboratory and cerebrospinal fluid (CSF) parameters were calculated.

**Results:** In total, 396 patients treated with an EVD were included into the study with a mean age of 54.3 (range: 18–89) years. EVD associated infections were observed in 32 (8.1%) patients. EVD insertion at another hospital (OR 3.86), and an increased CSF sampling frequency of more than every third day (OR 12.91) were detected as major risk factors for an EVD associated infection. The indication for EVD insertion, surgeon's experience, the setting of EVD insertion (ICU vs. operating room) and the operating time did not show any significant differences between the two groups. Furthermore, ROC analysis showed that clinical, serum laboratory and CSF parameters did not provide specific prediction of EVD associated infections (specificity 44.4%). This explains the high overtreatment rate in our cohort with the majority of our patients who received intrathecal vancomycin (63.3%), having either negative microbiological results (*n* = 12) or were defined as contaminations (*n* = 7).

**Conclusions:** Since clinical parameters and blood analyzes are not very predictive to detect EVD associated infections in neurosurgical patients, sequential but not too frequent microbiological and laboratory analysis of CSF are still necessary. Furthermore, we propose a uniform classification for EVD associated infections to allow comparability between studies and to sensitize the treating physician in determining the right treatment.

## Introduction

External ventricular drain (EVD) insertion is considered as the initial lifesaving treatment in many neurosurgical intensive care patients. Besides this therapeutic application, the continuous monitoring of the intracranial pressure (ICP) facilitated by the EVD remains one of the most important parameters for subsequent treatment decisions. While continuous cerebrospinal fluid (CSF) drainage may prevent further damage to the brain parenchyma from increased ICP, EVDs are associated with a high incidence of infections. Reported rates of EVD associated infections range between 0 and 23.2% (1–5) and lead to prolonged hospitalization of patients at intensive care units (ICU) (6). The number of studies investigating possible risk factors that lead to EVD associated infections are sparse and show divergent results (5–11). Indication for EVD insertion, multiple catheters used, duration of catheterization, CSF sampling frequency, and co-infection are usually described as the most common risk factors leading to EVD associated infections (5–9). However, comparison between studies is often complicated due to different definitions of EVD associated infections (2, 3, 10–12).

Historical studies described EVD associated infections as a single positive CSF culture (13–15) while more recent studies consider CSF pleocytosis, low glucose levels, and high protein levels as important additional parameters to the microbiological analysis in defining EVD associated infections (8, 16). Since CSF cell counts may report artificially high results due to blood in the ventricular system, Pople et al. recently proposed CSF pleocytosis with a white/red blood cell CSF count >0.02 as more accurate parameter for CSF infection (17).

Only a few historical studies have included the pathogen into their definition of EVD associated infection (4, 18), with recent neurosurgical studies neglecting this important factor into their definitions (3, 10, 11, 19). Non-neurosurgical studies have shown a variable pathogenicity between different bacterial species, especially in coagulase-negative staphylococci (CoNS) (20, 21), which is the predominant bacterial species causing EVD associated infections (3). Since a unified definition of EVD associated infection is currently not available, appropriate sample regimen and treatment measure protocols are missing as well. Therefore, we conducted this retrospective investigation of risk factors for EVD associated infections and used a modified practice-oriented definition of EVD associated infections based on the definition described by Schade et al. (18) and Hoefnagel et al. (4) (Table 1).

**Table 1:** Definitions of EVD-associated infections.

	**Schade et al. (18)**	**Hoefnagel et al. (4)**
ED-BM	- One positive CSF culture - Clinical signs of bacterial meningitis (fever, headache, nuchal rigidity, and/or altered mental status)	- Positive CSF-culture
Colonization	- Two or more positive CSF cultures with the same pathogen - No clinical signs	
Contamination	- Only one positive CSF culture with a common skin pathogen	- Only one positive CSF culture for a common skin pathogen - Negative consecutive samples

*CSF, cerebrospinal fluid; ED-BM, external drainage-related bacterial meningitis; EVD, external ventricular drainage*.

## Materials and Methods

### Ethical Approval

The study protocol was approved by the local Ethics Committee of the Medical University of Vienna (EK 1731/2019). For this type of study, formal consent is not required.

### Patient Cohort

We performed a retrospective analysis of patients treated with an EVD at the neurosurgical ICU of the Medical University of Vienna between 2008 and 2019. We included patients with at least one postoperative CSF sample at our department, independent of whether they were subsequently discharged, transferred to another hospital or died. The exclusion criteria were as follows: (1) Patients <18 years at the time of EVD insertion (2) duration of catheterization <24 h and (3) meningitis or ventriculitis prior to EVD insertion.

We gathered information on the following parameters: patients characteristics (age, sex), clinical, and laboratory infection markers of patients with EVD associated infection (fever, C-reactive protein, serum glucose, white blood cell (WBC) count), EVD implantation at another hospital, EVD implanted at the operating room vs. ICU, indication for EVD insertion [Subarachnoid hemorrhage (SAH), Tumor, Trauma, Intracerebral hemorrhage (ICH) or other], surgeons experience (consultant vs. resident), operating time, postoperative prophylactic antibiotic administration, treated systemic infection, number of needed EVD implantations, CSF sampling frequency during the total duration of catheterization, and prior to the diagnosis of an EVD associated infection, the total duration of catheterization, and the duration of catheterization prior to an EVD associated infection.

### External Ventricular Drainage System

EVD insertion was performed by a neurosurgical consultant or resident in the operating room or at the ICU under sterile conditions. The procedures were performed under appropriate sedation and analgesia with the patient in the supine position and the head of the bed elevated by 30–40 degrees. The area of insertions was shaved and prepared under sterile conditions. After identifying Kocher's point, a 2 cm straight incision at the pupilar line was performed. A self-retaining retractor was placed, and a small burr hole was drilled. After coagulating and incising the dura, a regular non-antibiotic coated ventricular catheter (Straight Ventricular Catheter F8, Integra®) was passed toward the ipsilateral medial canthus and ipsilateral tragus. Subsequently, the catheter was tunneled under the galea and secured by multiple sutures. Postprocedural prophylactic antibiotics were administered depending on the surgeon's preference with no standard protocol. At our department we usually use cefuroxime as prophylactic antibiotic. If clinically justifiable the EVD was weaned and finally clamped in the following days. Subsequently, if there were no neuroradiological signs of abnormally increased ventricular size, the clamped EVD was removed.

### CSF-Sampling Protocol

CSF-sampling was performed by a neurosurgical resident every other day, if feasible. After discarding the first ml of CSF, two ml were withdrawn from the EVD to investigate CSF cell count, protein, glucose, and lactate levels and 4 ml of CSF were sampled for the microbiological analysis. CSF withdrawal was performed under sterile conditions from an outlet, which is around 30 cm distal to the EVD insertion.

### Clinical Definitions

Based on the definition at our local neurosurgical ICU, fever was defined as body temperature ≥ 37.5°C, measured by a urinary catheter. For data analysis, the highest measured temperature-value at the day of infection was used. Antibiotic administration was considered prophylactic, if administration was performed within 24 h after EVD insertion with no signs of infection. Treated systemic infection was defined as antibiotic treatment due to a systemic infection, such as pneumonia, urinary tract infection, or sepsis with positive blood cultures.

Based on the reference values of our local laboratory, normal CSF was defined as a cell count of <4 per μl, protein of 15–45 mg/dL, lactate <2.1 mmol/L, and glucose > 50% of systemic glucose. Since most patients with EVDs suffer from intraventricular hemorrhage, CSF values show abnormal results even in the absence of an EVD associated infection. Hence, CSF values alone were not used as parameters to identify an EVD associated infection.

### Treatment Protocol of EVD Associated Infections

According to our local management guidelines, EVDs were exchanged in cases of a suspected infection, and systemic antibiotic therapy was started according to the corresponding antibiogram or empirically if an antibiogram was absent. Subsequently, CSF sampling frequency was increased to determine the efficacy of the antibiotic therapy.

Intrathecal antibiotic therapy with vancomycin was applied twice daily for 7 days if: CSF parameters did not improve after systemic antibiotic treatment and (1) an infection was highly suspected in the absence of a positive microbiological culture or (2) microbiological analysis showed gram-positive bacteria. Suspicion of infection was given in cases of continuously increasing CSF cell count, lactate and body temperature and a decreasing CSF/Serum Glucose ratio.

### Definition of Infections and Retrospective Classification

Due to the lack of a uniform definition concerning EVD associated infections and the variable pathogenicity between different bacterial species, we decided to use a modified definition provided by Schade et al. (18) and Hoefnagel et al. (4). In consensus with the Division of Infectious Diseases and Tropical Medicine of the Medical University of Vienna we decided to retrospectively categorize each patient based on their microbiological CSF results as summarized in Table 2. The allocation was performed based on the consensus of one neurosurgeon (F.K.) and one infectiologist (M.G.V.). Patients from the “infection”-group and those with negative microbiological results who received intrathecal vancomycin were considered as patients with “suspected infections.”

**Table 2:** Modified classification of EVD associated infection.

No-infection	Negative microbiological results	• Negative CSF cultures
	Contamination	• An isolated positive CSF culture with coagulase negative Staphylococci or *Cutibacterium acnes* with a time to positivity > 24 h
Infection	Colonization	• Multiple positive CSF cultures with coagulase negative Staphylococci or Cutibacteria with a time to positivity within 15 h
	Ventriculitis	• Colonization in combination with an increased C-reactive protein at the time of CSF-sampling • Single or multiple positive CSF-cultures containing *Enterobacter cloacae, Staphylococcus aureus, Serratia spp., Streptococcus spp*. and/or *Enterococcus faecalis*

*CSF, cerebrospinal fluid*.

### Statistical Analysis

Statistical analysis was performed with SPSS software version 26.0 (IBM, Armonk, NY, USA). Normal distributed values were expressed as mean ± standard deviation and analyzed by unpaired student *t*-test. Non-parametric values were analyzed by Mann-Whitney-*U*-test. For the analysis of contingency tables Chi-square test or Fisher's exact test for small sample sizes were used. Significant differences between nominal or ordinal variables were expressed as odds ratio (OR) and their 95% confidence intervals (95% CI). To investigate the predictive power of body temperature and serum infection parameters as well as body temperature and combined serum and CSF markers for the presence of an actual EVD infection, binary regression models were conducted for all cases of suspected infections and the derived prediction formulas were tested using ROC models to define the most meaningful cutoff values according to sensitivity and specificity values. Due to the explorative character of this study correction for multiple testing was not used and a *p* < 0.05 was considered as statistically significant for all performed tests.

## Results

**Figure 1:**
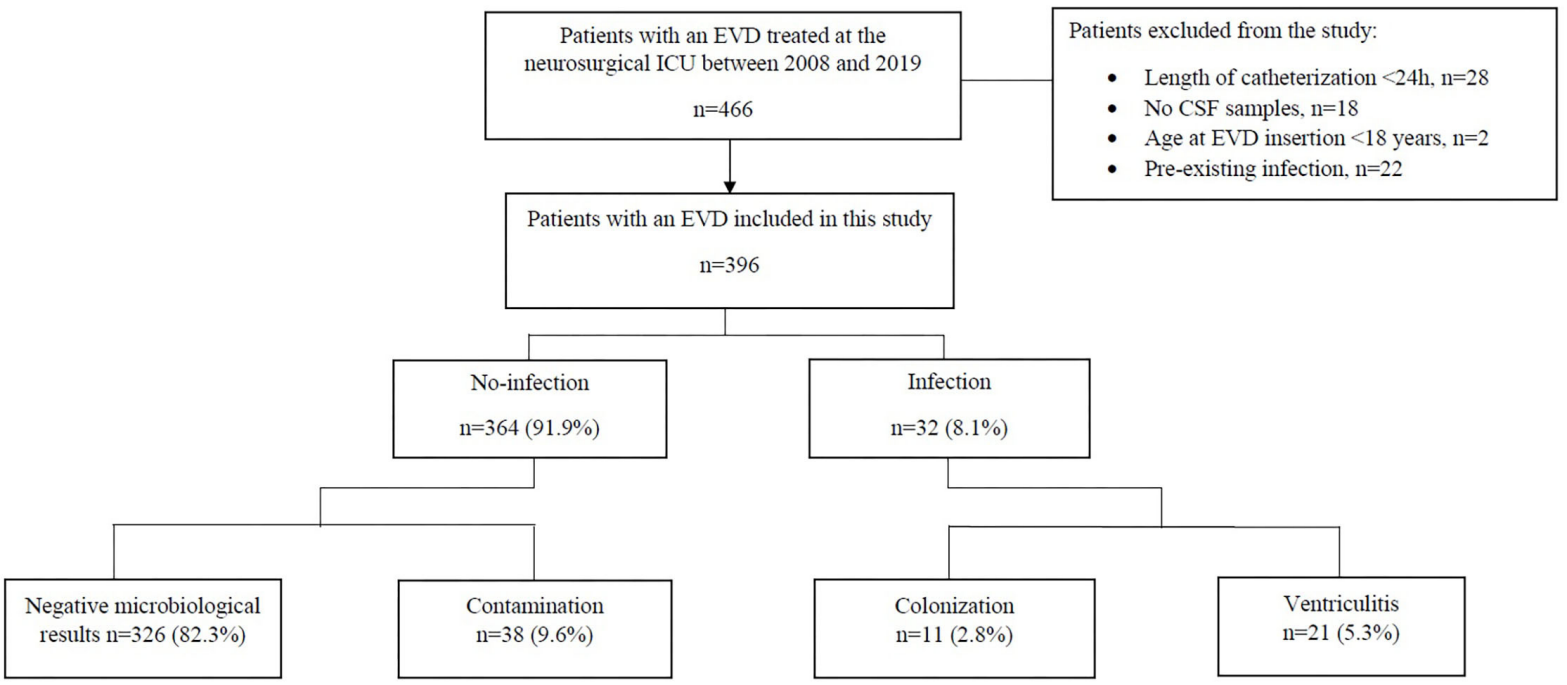
Flow chart showing the study inclusion algorithm. CSF, cerebrospinal fluid; EVD, external ventricular drainage; ICU, intensive care unit.

We analyzed the data of 396 patients treated by EVD at a mean age of 54.3 years (range: 18–89) as illustrated in Figure 1. Patient characteristics and differences between patients with and without EVD infection are shown in Table 3. There were 220 (55.6%) female patients. 17 (4.29%) patients received their first EVD from another hospital, with five of these patients receiving a bolt-connected EVD. The indications for EVD placement were SAH in 262 (66.2%), ICH in 68 (17.2%), traumatic brain injury in eight (2%), obstructive hydrocephalus due to tumor progression in 43 (10.9%) and others in 15 (3.8%). Communicating hydrocephalus of unknown origin was summarized as “others.” 171 (43.2%) and 225 (56.8%) patients received their first EVD in the operating room and at the ICU, respectively.

**Table 3:** Differences in patient characteristics and EVD management between patients with and without EVD associated infections.

**Variable**	**Number of patients (%)**		
	**Infection**	**No-infection**	***P*-value**
Total number of patients (*n =* 396)	32 (8.1%)	364 (91.9%)	
**Sex**			
Male (*n =* 176)	16 (50%)	160 (44%)	NS
Female (*n =* 220)	16 (50%)	204 (56%)	NS
Mean age in years (range)	52.9 (20–73)	54.4 (18–89)	NS
**EVD from another hospital (*****n** **=*** **17)**	**4 (12.5%)**	**13 (3.6%)**	0.04^*^
EVD implantation at			
OR (*n =* 171)	13 (40.6%)	155 (42.6%)	NS
ICU (*n =* 225)	19 (59.4%)	209 (57.4%)	NS
Indication for EVD			
SAH (*n =* 262)	22 (68.8%)	240 (65.9%)	NS
Tumor (*n =* 43)	2 (6.3%)	41 (11.3%)	NS
Trauma (*n =* 8)	0	8 (2.2%)	NS
ICH (*n =* 68)	8 (25%)	60 (16.5%)	NS
Other (*n =* 15)	0	15 (4.1%)	NS
Surgeon's experience			
Consultant (*n =* 198)	13 (40.6%)	185 (50.8%)	NS
Resident (*n =* 198)	19 (59.4%)	179 (49.2%)	NS
Type 2 diabetes (*n =* 38)	1 (3.1%)	37 (10.2%)	NS
Immunosuppression or use of steroids (*n =* 47)	5 (15.6%)	42 (11.5%)	NS
Mean operating time in minutes (SD)	50.9 (53.6)	52.6 (57.3)	NS
Mean operating time of EVD insertion alone in minutes (SD)	35.2 (15)	37.3 (16.2)	NS
Postoperative prophylactic antibiotics (*n =* 349)	26 (81.3%)	323 (88.7%)	NS
Treated systemic infection (*n =* 300)	25 (78.1%)	275 (75.5%)	NS
Multiple EVDs at EVD insertion (*n =* 37)	4 (12.5%)	33 (9.1%)	NS
**Multiple EVDs during total period of catheterization (*****n** **=*** **103)**	**18 (56.3%)**	**85 (23.4%)**	<0.001^***^
Number of EVDs before infection (range)	1.5 (1–4)	1.4 (1–4)	NS
**Mean CSF sampling frequency during the total period of EVD treatment (range)**	**12.2 (2–52)**	**6.8 (1–25)**	<0.001^***^
Mean CSF sampling frequency before infection (range)	7.1 (1–30)	6.8 (1–25)	NS
**Mean ratio of sampling frequency and duration of catheterization before infection (range)**	**0.6 (0.17–1)**	**0.46 (0.1–1.17)**	<0.001^***^
**Mean duration of catheterization in days (range)**	**25.6 (7–163)**	**15.7 (2–55)**	0.012^*^
Mean duration of catheterization of first EVD in days (range)	14 (1–70)	12.6 (0–55)	NS
**Reinsertion frequency after first EVD during total period of catheterization (range)**	**1.7 (1–4)**	**1.1 (1–3)**	<0.001^***^

*Values are expressed as numbers (%) or as mean (range)*.

*CSF, cerebrospinal fluid; EVD, external ventricular drainage; ICH, intracerebral hemorrhage; ICU, intensive care unit; NS, not significant; OR, operating room; SAH, subarachnoid hemorrhage; SD, standard deviation*.

* *p ≤ 0.05*,

*** *p ≤ 0.001*.

*Statistically significant values are in bold*.

EVDs were inserted by residents as frequently (50%) as by consultants. The mean operating time was 52.5 min. In some cases, EVD insertion was performed as part of another operation (e.g., aneurysm clipping). Therefore, the time of EVD insertion alone was not available in some cases. By excluding these cases, the mean operating time of EVD insertion alone was 37.1 min.

All patients received at least a single dose of a periprocedural prophylactic antibiotic. Depending on the surgeon's preference 350 (88.4%) patients received further postoperative prophylactic antibiotics over 3 days. 300 (75.8%) patients had an antibiotic treatment due to a systemic infection during their stay at the ICU.

According to their CSF culture findings, patients were retrospectively classified into EVD associated infections (*n* = 32) with its two subgroups ventriculitis 21 (5.3%) and colonization 11 (2.8%), and no-infections (*n* = 364) consisting of 38 (9.6%) contaminations and 326 (82.3%) negative microbiological results (Table 4).

**Table 4:** Characteristics of the study patients.

**Variable**	**Number of patients (%)**			
	**Infection**		**No-infection**	
	**Ventriculitis**	**Colonization**	**Contamination**	**Negative microbiological results**
Total number of patients (*n =* 396)	21 (5.3%)	11 (2.8%)	38 (9.6%)	326 (82.3%)
**Sex**				
Male (*n =* 176)	11 (52.4%)	5 (45.5%)	14 (36.8%)	146 (44.8%)
Female (*n =* 220)	10 (47.6%)	6 (54.5%)	24 (63.2%)	180 (55.2%)
Age (in years)—mean (range)	51.3 (20–70)	56 (37–73)	54.6 (30–72)	54.4 (18–89)
EVD from another hospital (*n =* 17)	2/21	2/11	1/38	12/326
**Room of procedure**				
OR (*n =* 171)	7 (33.3%)	6 (54.5%)	13 (34.2%)	142 (43.6%)
ICU (*n =* 225)	14 (66.7%)	5 (45.5%)	25 (65.8%)	184 (56.4%)
**Indication for EVD**				
SAH (*n =* 262)	13 (61.9%)	9 (81.8%)	28 (73.7%)	212 (65%)
Tumor (*n =* 43)	2 (9.5%)	0	4 (10.5%)	37 (11.3%)
Trauma (*n =* 8)	0	0	1 (2.6%)	7 (2.1%)
ICH (*n =* 68)	6 (28.6%)	2 (18.2%)	5 (13.2%)	55 (16.9%)
Other (*n =* 15)	0	0	0	15 (4.6%)

*Values are expressed as numbers (%) or as mean (range)*.

*EVD, external ventricular drainage; ICH, intracerebral hemorrhage; ICU, intensive care unit; OR, operating room; SAH, subarachnoid hemorrhage*.

Concerning age, sex, the indication for EVD insertion and the amount of EVDs used before infection, no significant differences were found between the infection and no-infection groups. Furthermore, the setting of EVD placement (ICU vs. operating room), the surgeons experience (consultant vs. resident), the operating time, postoperative prophylactic antibiotic administration and systemic infection did not influence the occurrence of EVD associated infections (Table 3).

### Factors Related to EVD Associated Infections

As a tertiary referral center, we provide endovascular and open neurosurgical treatment options for patients with neurovascular diseases. Thus, we regularly admit patients with a pre-inserted CSF-drainage from their on-site neurosurgical treatment center. Thus, 17 out of 396 (4.3%) patients were transferred to our department with an EVD already in place. Here we could show that these patients had a significantly higher risk to develop an EVD associated infection (12.5 vs. 3.6%, *p* = 0.04^*^) with an OR of 3.86 (1.2–12.6) (Table 3). Two of the 5 patients who received a bolt-connected EVD, had an EVD associated infection. However, the bolt-system did not have a statistically significant increased risk in promoting an EVD associated infection, compared to our conventional approach of EVD insertion (*p* = 0.054).

There was no significant difference of the CSF sampling frequency between the infection and no-infection groups prior to the diagnosis of an EVD infection. However, by comparing the ratio between the CSF sampling frequency and the duration of catheterization prior to the diagnosis of an EVD infection, a significant difference (0.6 vs. 0.46 CSF samplings/day, *p* < 0.001^***^) could be observed, with a much higher ratio in the infection group. Patients with >0.33 CSF samplings/day had a significantly increased risk of developing an EVD associated infection (*p* < 0.001^***^), with an OR of 12.91. As assumed, the CSF sampling frequency was significantly higher in the infection group during the total period of EVD treatment (12.2 vs. 6.8, *p* < 0.001^***^).

By comparing the number of total EVD reinsertions needed per patient, we could observe a significantly increased number in the infection group, compared to the no-infection group (mean, 1.7 vs. 1.1, *p* < 0.001^***^). The period of catheterization with the first EVD did not show a significant difference between the two groups (infection vs. no-infection, 14 vs. 12.6 days, NS). However, the total duration of catheterization was significantly prolonged in the infection group, compared to the no-infection group (25.6 vs. 15.7 days, *p* = 0.012^*^).

### Microbiology and Systemic Infection

*Staphylococcus spp*. (75%) was most common, with *Staphylococcus epidermidis* (45%) being the most frequent species, followed by *Staphylococcus haemolyticus* (13.8%). There were 3 cases of *Staphylococcus aureus* (3.6%) infections.

10/32 (31.3%) patients from the infection group and 10/19 (52.6%) patients from the no-infection group who received intrathecal vancomycin treatment had no systemic infection at the time of positive CSF culture or start of intrathecal vancomycin treatment (Table 5, Supplementary Table 1).

**Table 5:** Characteristics of patients with EVD associated infections.

**Patients**	**Categories based on microbiological findings**	**Gram stain**	**Species**	**CSF cell count (per μl)**	**Systemic infection at the time of pos. CSF culture**	**Intrathecal vancomycin**	**Postoperative prophylactic antibiotic**
1	Ventriculitis		*Candida albicans*	5	No systemic infection		No
2	Ventriculitis		*Candida albicans*	1,500	Pneumonia		
3	Ventriculitis	Negative	*Enterobacter cloacae*	833	Pneumonia		
4	Ventriculitis	Positive	*Streptococcus parasanguinis*	400	Pneumonia		
5	Ventriculitis	Both	*Neisseria sicca, Staphylococcus mitis, Staphylococcus hominis*	404	Pneumonia		No
6	Ventriculitis	Positive	*Nocardia cyriacigeorgica*	347	No systemic infection	Yes	
7	Ventriculitis	Positive	*Staphylococcus aureus*	28	Urinary tract infection, Pneumonia	Yes	
8	Ventriculitis	Positive	*Enterococcus faecalis*	171	No systemic infection	Yes	
9	Ventriculitis	Positive	*Kocuria species*	27	No systemic infection	Yes	
10	Ventriculitis	Positive	*Staphylococcus aureus*	327	Pneumonia	Yes	
11	Ventriculitis	Positive	*Staphylococcus aureus, MRSA*	1,648	Pneumonia, Urinary tract infection, pos. blood cultures	Yes	No
12	Ventriculitis	Positive	*Streptococcus oralis*	25,020	Urinary tract infection	Yes	
13	Ventriculitis	Positive	*Enterococcus faecalis*	5	Pneumonia	Yes	
25	Ventriculitis	Negative	*Acinetobacter*	69	Urinary tract infection		
26	Ventriculitis	Positive	*Staphylococcus haemolyticus*	139	No systemic infection		
27	Ventriculitis	Positive	*Staphylococcus epidermidis*	47	No systemic infection		
28	Ventriculitis	Positive	*Staphylococcus epidermidis, Staphylococcus pettenkoferi, Staphylococcus caprae, Corynebacterium tuberculostearicum*	38	Pneumonia		
29	Ventriculitis	Positive	*Staphylococcus epidermidis*	11,264	Urinary tract infection		
30	Ventriculitis	Negative	*Serratia marcescens*	3	Pneumonia		
31	Ventriculitis	Positive	*Staphylococcus epidermidis*	75	Urinary tract infection	Yes	
32	Ventriculitis	Positive	*Viridans streptococci*	54	No systemic infection		
14	Colonization	Positive	*Staphylococcus epidermidis*	274	No systemic infection	Yes	
15	Colonization	Positive	*Staphylococcus epidermidis*	46	Pneumonia	Yes	No
16	Colonization	Positive	*Staphylococcus haemolyticus*	66	Pseudomonas sepsis		No
17	Colonization	Positive	*Staphylococcus epidermidis*	33	Urinary tract infection, Pneumonia		
18	Colonization	Positive	*Staphylococcus epidermidis*	39	Urinary tract infection		
19	Colonization	Positive	*Staphylococcus haemolyticus*	53	Urinary tract infection		
20	Colonization	Positive	*Staphylococcus epidermidis*	31	Pneumonia		No
21	Colonization	Positive	*Staphylococcus haemolyticus*	29	No systemic infection		
22	Colonization	Positive	*Staphylococcus epidermidis, Staphylococcus hominis*	26	No systemic infection		
23	Colonization	Positive	*Staphylococcus epidermidis*	174	Urinary tract infection		
24	Colonization	Positive	*Staphylococcus epidermidis*	51	Urinary tract infection		

*CSF, cerebrospinal fluid; EVD, external ventricular drainage*.

### Intrathecal Vancomycin Application

Intrathecal vancomycin treatment was applied in 30 patients of which 11 (36.7%) were classified as infections and 19 (63.3%) as no-infections. All microbiological and laboratory characteristics of the infection group are summarized in Table 5, Supplementary Table 1. Nineteen patients from the no-infection group received intrathecal vancomycin therapy, despite microbiological analysis of CSF revealing only contamination (*n* = 7) or no signs of infection (*n* = 12) (Supplementary Table 1). All 12 patients with negative microbiological results showed an increased CSF cell count with a mean of 1,558 cells/μl (± 1,270 cells/μl) and elevated CSF lactate values with a mean of 4.4 mmol/L (± 1.5 mmol/l), respectively. The CSF/serum glucose ratio was decreased in 8 of these 12 patients with a mean of 0.43 (range: 0.16–0.62) and fever occurred in 8 patients.

### Laboratory Parameters

To evaluate whether EVD related infections could be detected prior to the results of the microbiological analysis, we assessed various clinical, and laboratory parameters at the day when the positive CSF sample was taken. The results are summarized in Supplementary Table 2. The mean CSF cell count of EVD infections was 1,351 cells/μl (± 4,755 cells/μl) with 31 of 32 (96.9%) patients having an increased cell count > 4 cells/μl. The mean CSF-lactate was 3.7 mmol/L (range: 1.4–11 mmol/L) with 25 (78.1%) patients having an increased CSF lactate value > 2.1 mmol/L. Fever occurred in 24 (75%) patients.

The initial regression model including all available clinical infection markers not requiring CSF sampling was based on data of 11 (36.7%) patients with correctly suspected CSF infections and 19 (63.3%) patients with incorrectly suspected CSF infections. By including body temperature and serum markers at the time of suspected infection, the following formula was established:


$$\begin{array}{l} 0.564^{*}\text{temperature}+0.093^{*}\text{WBC}+0.022^{*}\text{CRP}\\ +\;0.022^{*}\text{serum\_glucose}. \end{array}$$

**Figure 2:**
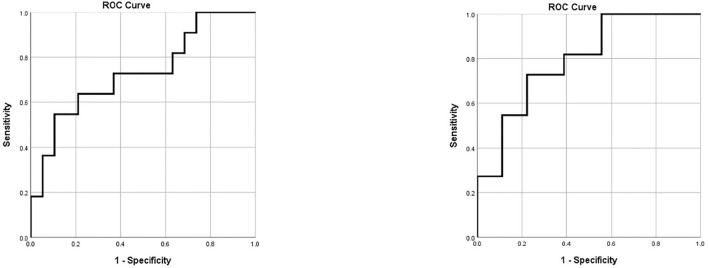
ROC curves for the prediction of EVD associated infections. Left: ROC curve for the prediction of EVD associated infection according to body temperature and blood serum markers alone. Right: ROC curve for the prediction of EVD associated infection according to body temperature, blood serum markers and CSF parameters. CSF, cerebrospinal fluid; EVD, external ventricular drainage; ROC, receiver operating characteristics.

The ROC model demonstrated a sensitivity of 100% and a specificity of 26.3% for the detection of an actual infection at a threshold of 24.7754 (Figure 2).

The subsequently performed regression model additionally including CSF markers included data of 11 (27.9%) patients with correctly suspected CSF infections and 18 (62.1%) patients with incorrectly suspected CSF infections and yielded the following formula:


$$\begin{array}{l} 0.835^{*}\text{temperature}+0.192^{*}\text{WBC}+0.062^{*}\text{CRP}\\ +\;0.021^{*}\text{serum\_glucose}+0.000^{*}\text{cellcount}\\ +\;0.064^{*}\text{CSF\_glucose}+0.000^{*}\text{CSF\_protein}\\ +\;0.065^{*}\text{CSF\_lactate}. \end{array}$$

In one patient CSF values were not available at the time of microbiological analysis due to insufficient CSF-sampling.

The ROC model for this more precise predication formula demonstrated a sensitivity of 100% and a specificity of 44.4% for the detection of an actual infection at a threshold value of 40.6039 (Figure 2).

### Patients With and Without Postoperative Prophylactic Antibiotic Treatment

Since postoperative prophylactic antibiotic treatment following a periprocedural single shot antibiotic administration may affect factors that are associated with EVD infections, we consequently separated our patients into the following two groups: (1) Patients with postoperative prophylactic antibiotic treatment (*n* = 349) and (2) patients without postoperative prophylactic antibiotic treatment (*n* = 47). The results are summarized in Table 6. By excluding patients that did not receive postoperative prophylactic antibiotic treatment, we could show that all previously significant results remained significant. In contrast, by analyzing only patients without postoperative prophylactic antibiotics, we could not find any significant results in this small cohort.

**Table 6:** Differences in patient characteristics and EVD management between patients with and without EVD associated infections.

**Variable**	**Number of patients with postoperative prophylactic antibiotic therapy**, ***n** **=*** **349 (%)**			**Number of patients without postoperative prophylactic antibiotic therapy**, ***n** **=*** **47 (%)**		
	**Infection**	**No-infection**	***P*-value**	**Infection**	**No-infection**	***P*-value**
Total number of patients (*n =* 396)	26 (7.4%)	323 (92.6%)		6 (12.8%)	41 (87.2%)	
**Sex**						
Male (*n =* 176)	11 (42.3%)	139 (43%)	NS	5 (83.3%)	21 (51.2%)	NS
Female (*n =* 220)	15 (57.7%)	184 (57%)	NS	1 (16.7%)	20 (48.8%)	NS
Mean age in years (range)	54.4 (25–73)	54.4 (18–88)	NS	46.7 (20–68)	54.5 (24–89)	NS
**EVD from another hospital (*****n** **=*** **17)**	**4 (15.4%)**	**13 (4%)**	0.03^*^	0	0	
EVD implantation at						
OR (*n =* 171)	11 (42.3%)	138 (42.7%)	NS	2 (33.3%)	17 (41.5%)	NS
ICU (*n =* 225)	15 (57.7%)	185 (57.3%)	NS	4 (66.7%)	24 (58.5%)	NS
Indication for EVD						
SAH (*n =* 262)	18 (69.2%)	213 (65.9%)	NS	4 (66.7%)	27 (65.9%)	NS
Tumor (*n =* 43)	1 (3.8%)	36 (11.1%)	NS	1 (16.7%)	5 (12.2%)	NS
Trauma (*n =* 8)	0	8 (2.5%)	NS	0	0	
ICH (*n =* 68)	7 (26.9%)	51 (15.8%)	NS	1 (16.7%)	9 (22%)	NS
Other (*n =* 15)	0	15 (4.6%)	NS	0	0	
Surgeon's experience						
Consultant (*n =* 198)	10 (38.5%)	163 (50.5%)	NS	3 (50%)	22 (53.7%)	NS
Resident (*n =* 198)	16 (61.5%)	160 (49.5%)	NS	3 (50%)	19 (46.3%)	NS
Type 2 diabetes (*n =* 38)	1 (3.8%)	34 (10.5%)	NS	0	3 (7.3%)	NS
Immunosuppression or use of steroids (*n =* 47)	4 (15.4%)	35 (10.8%)	NS	1 (16.7%)	7 (17.1%)	NS
Mean operating time in minutes (SD)	49.1 (53.6)	53.9 (59.5)	NS	59 (59.5)	42.5 (34.1)	NS
Mean operating time of EVD insertion alone in minutes (SD)	35.7 (16.2)	37.5 (16.6)	NS	32.5 (6.5)	35.5 (12.7)	NS
Treated systemic infection (*n =* 300)	19 (73.1%)	244 (75.5%)	NS	6 (100%)	31 (75.6%)	NS
Multiple EVDs at EVD insertion (*n =* 37)	4 (15.4%)	29 (9%)	NS	0	4 (9.8%)	NS
**Multiple EVDs during total period of catheterization (*****n** **=*** **103)**	**15 (57.7%)**	**76 (23.5%)**	<0.001^***^	3 (50%)	9 (22%)	NS
Number of EVDs before infection (range)	1.6 (1–4)	1.4 (1–4)	NS	1	1.3 (1–2)	NS
**Mean CSF sampling frequency during the total period of EVD treatment (range)**	**11 (2–34)**	**6.9 (1–25)**	0.008**	17.2 (4–52)	6.1 (1–19)	NS
Mean CSF sampling frequency before infection (range)	6.9 (1–19)	6.9 (1–25)	NS	7.7 (1–30)	6.1 (1–19)	NS
**Mean ratio of sampling frequency and duration of catheterization before infection (range)**	**0.57 (0.17–1)**	**0.46 (0.1–1.17)**	0.002**	0.75 (0.42–1)	0.45 (0.13–0.75)	NS (0.05)
**Mean duration of catheterization in days (range)**	**22.2 (7–51)**	**15.9 (2–55)**	0.021*	40.3 (7–163)	13.9 (2–34)	NS
Mean duration of catheterization of first EVD in days (range)	12.9 (1–27)	12.8 (0–55)	NS	19 (5–70)	11.1 (1–34)	NS
**Reinsertion frequency after first EVD during total period of catheterization (range)**	**1.7 (1–4)**	**1.1 (1–3)**	0.038*	1.7 (1–2)	1	NS

*Values are expressed as numbers (%) or as mean (range)*.

*CSF, cerebrospinal fluid; EVD, external ventricular drainage; ICH, intracerebral haemorrhage; ICU, intensive care unit; NS, not significant; OR, operating room; SAH, subarachnoid haemorrhage; SD, standard deviation*.

* *p ≤ 0.05*,

*** *p ≤ 0.001*.

*Statistically significant values are in bold*.

## Discussion

Multiple risk factors have been described to be associated with EVD associated infections (4–9, 22), with prolonged duration of catheterization (4, 5, 7, 8, 15, 17, 22) and increased CSF-sampling frequency (4, 23) being considered as the most likely causes. However, it remains ambiguous if these factors are the consequences of EVD associated infections or if they promote infections. By separately observing the CSF sampling frequency and duration of catheterization before infections occurred, our results showed no significant differences between the infection and no-infection groups. These factors, however, were significantly increased during the total duration of catheterization. To prevent wrong assumptions, we adjusted the CSF sampling frequency to the duration of catheterization. With the introduction of a CSF sampling/day ratio, we could show that the development of EVD associated infections was significantly higher if CSF samplings were performed more than every third day (>0.33 sampling/day, OR 12.91). Consequently, patients with an EVD associated infection showed a significantly higher number of needed EVDs and a significantly prolonged total duration of catheterization with an increased total CSF sampling frequency. According to our local management guidelines, EVDs of patients with an EVD associated infection were exchanged at the time of the positive microbiological result. Subsequently, these patients received concomitant antibiotics according to the corresponding antibiogram with intrathecal vancomycin in some cases. To evaluate the therapeutic outcome, we adhered to our CSF sampling frequency protocol, which led to an increased duration of catheterization.

Furthermore, it should be noted that while we intended to perform CSF-samplings every other day, certain situations have led to a variation in CSF sampling frequencies. In cases of slit ventricles or obstruction of the EVD system, CSF sampling was hindered, which led to a reduced CSF sampling frequency. In contrast, if an EVD associated infection was suspected, CSF sampling frequency was increased to observe and improve treatment measures.

Contrary to other studies (9, 24), we did not find a significant association between infections and the setting of EVD placement (ICU vs. operating room), but patients with a preinserted EVD from another hospital were significantly more prone to develop an EVD associated infection compared to patients who received their EVD at our department. Interestingly, patients with a bolt-connected EVD showed a high rate of EVD associated infections (2/5, 40%), however, we did not find a significant association between these two variables (*p* = 0.054). The retrospective design of this study and the small sample size of bolt-systems used, did not allow an appropriate comparison. Notwithstanding that it has been shown that bolt-kit EVD systems do not elevate the risk of EVD associated infections (25), we assume that infections occurred due to increased handling of the patients during the transfer process.

To expedite the detection of an EVD associated infection, we tried to investigate CSF parameters that have been inferred to EVD associated infections, as CSF-cell count and -lactate values (17, 26). By combining the results of CSF parameters, laboratory results, and clinical signs of infection to detect an EVD associated infection, the ROC curve showed a sensitivity of 100% and a specificity of 44.4%. Hence, we concur with other studies that these parameters alone are not specific enough to predict EVD associated infections. The high rate of false positives in patients with SAH or ICH (27, 28) emphasize the need for more sensitive diagnostic CSF markers. Furthermore, to reduce our CSF sampling frequencies, we investigated only laboratory and clinical parameters that could infer to EVD associated infections. Again, we could not define any parameter that predicted an EVD associated infection with a high specificity (sensitivity 100%, specificity 24.8%). This low specificity of clinical signs and laboratory parameters was already observed by Schade et al. (18).

The inaccurate suspicion of an EVD associated infection could lead to non-adequate treatment measures. In retrospect, the majority of our patients who received intrathecal vancomycin treatment showed either negative microbiological results (*n* = 12) or were defined as contaminations (*n* = 7). The intrathecal vancomycin application in cases of a suspected infection with the aim of CSF sterilization has been historically embedded at our department and used off label. To our knowledge, there are no studies which evaluated the intrathecal vancomycin treatment in suspected infections. However, studies have shown that the intrathecal vancomycin application in health care associated ventriculitis is well-tolerated and leads to a high number of CSF sterilization (68–88.4%) (29, 30) with better pharmacodynamics and similar efficacy and safety compared to intravenous antimicrobial agents (31–33). Most of our patients with incorrectly suspected infections received an EVD after SAH or ICH (*n* = 17/19) and showed an increased CSF cell count with a decreased CSF glucose. These patients did not have any long-term consequences regarding vancomycin application. Nevertheless, their duration of catheterization was equal to patients who had a positive CSF culture. Therefore, overtreatment has led to a longer duration of catheterization.

Furthermore, we acknowledge that most of our patients received postoperative prophylactic antibiotics (88.4%) and therefore the detection of certain bacterial species may be suppressed. To overcome this limitation, we subsequently performed data analysis separately for both groups who received or did not receive postoperative prophylactic antibiotic treatment. In the group who received postoperative prophylactic antibiotic treatment the aforementioned significant results remained significant and therefore factors like “EVD from another hospital” and an increased “CSF sampling/day” ratio still have a great impact on promoting EVD associated infections. In contrast, we could not find any significant result in the group that did not receive postoperative prophylactic antibiotic treatment. However, this comparison is limited by a very small sample size in the “infection” group. We believe that the investigation of risk factors promoting infections would be improved by analyzing patients without any antibiotic treatment. Nonetheless, this scenario is hindered due to our clinical guidelines, which we believe has led to low EVD associated infection rates (8.1%) in our cohort. This is in accordance with a recently published meta-analysis by Sheppard et al., who showed that an extended systemic antibiotic treatment could lower the risk of ventriculostomy related infections to 3–9%. In contrast, with perioperative prophylactic antibiotic treatment the incidence rate of ventriculostomy related infection was 7–18% (19).

Finally, we want to emphasize the importance of a standardized definition of EVD associated infections. According to Hoefnagel et al. (4), former studies showed varying definitions of EVD associated infections, which is apparent in the wide range of infection rates, and were limited by a non-uniform assessment of clinical and laboratory parameters (2, 22, 23, 34). Due to these limitations, a comparison between studies and further verification of clinical and laboratory parameters is challenging. Furthermore, despite the accurate definition of meningitis and ventriculitis by the Center for Disease Control and Prevention (24, 35), neurosurgical ICU patients with an EVD should be considered separately due to the initial heterogenous alteration in CSF parameters after SAH or ICH. Therefore, microbiological CSF analysis is still needed and should be considered the most accurate method in diagnosing EVD associated infections, particularly in neurologically compromised patients. This study proposes the implementation of a slightly altered classification system (Table 2) based on Schade et al. (18) and Hoefnagel et al. (4) which is based on microbiological results, since different bacterial species show varying pathogenicity (20).

We suggest the following clinical approach in the management of EVDs: CSF samples should be routinely drawn twice a week for microbiological analysis or as soon as possible in cases of clinically suspected ventriculitis. No treatment measures are necessary in cases of contamination or if CSF cultures remain negative. If an EVD associated infection is detected according to our classification system, the EVD should be exchanged. In cases of colonization an antibiotic treatment should not be necessary. In cases of secondary ventriculitis an antibiotic treatment should be started according to the antibiogram. The duration of the antibiotic treatment should be between 7 and 21 days, based on the microbiological results and alterations of CSF parameters (33).

We believe that our modified practice-oriented definition of EVD associated infections could ensure an appropriate exchange of information and improve patient specific treatment measures. It should guide clinicians and prevent unnecessary EVD revision surgery as well as incorrect application of systemic and intrathecal antibiotic therapy.

## Strengths and Limitations

Due to its retrospective design this study has limitations that should be considered when interpreting our results. We acknowledge that infections are multi-factorial, and it is highly probable that undocumented factors, e.g., CSF leakage and wound healing have an impact on EVD associated infection rates. Additionally, since all our patients were treated at the intensive care unit most of them received antibiotics either postoperatively (88.4%) or due to a systemic infection (75.8%). The detection of certain bacterial species therefore may be suppressed. Finally, we want to emphasize that our results are specific to neurosurgical patients with a high rate of SAH (66.2%) patients, which may impact its external validity, especially for centers who mainly treat trauma patients. The strength of our study is that we continuously applied a standard CSF-sampling protocol over one decade with long observation periods and frequent CSF samplings that facilitated the detection of potential factors promoting EVD associated infections.

## Conclusions

EVD associated infections are confirmed by positive microbiological findings which are often not available immediately. Therefore, in cases of suspected infections physicians often make treatment decisions only based on clinical findings and laboratory results. But these parameters may be altered due to systemic infections as well as intraventricular hemorrhage in the critically ill. The present study showed that there is a lack of specific parameters that might predict EVD associated infections. Based on the risk factors identified in our study we believe that CSF sampling should still be performed routinely but with a reduced frequency to avoid iatrogenic infections. Thus, the sequential dynamic alterations of CSF parameters allow for an improved diagnosis of suspected EVD associated infections. However, if clinically justifiable, CSF parameters should never be used alone to predict EVD associated infections, but in combination with CSF cultures.

Finally, as it was emphasized by many authors before, a uniform definition of EVD associated infections is proposed that ensures that there is comparability between studies and, at the same time, supports the treating physician in making right treatment decisions.

## Data Availability Statement

The original contributions presented in the study are included in the article/Supplementary Material, further inquiries can be directed to the corresponding author/s.

## Ethics Statement

The studies involving human participants were reviewed and approved by Ethics Committee of the Medical University of Vienna (EK 1731/2019). Written informed consent for participation was not required for this study in accordance with the national legislation and the institutional requirements.

## Author Contributions

FK, NF, MM, MV, AR, CD, KR, and JH contributed to conception, design, and methodology of the study. FK, NF, and JH organized the database. FK, MM, and JH performed the statistical analysis. FK wrote the first draft of the manuscript. JH wrote sections of the manuscript and supervised the study. All authors contributed to manuscript revision, read, and approved the submitted version.

## Conflict of Interest

The authors declare that the research was conducted in the absence of any commercial or financial relationships that could be construed as a potential conflict of interest.

## Publisher's Note

All claims expressed in this article are solely those of the authors and do not necessarily represent those of their affiliated organizations, or those of the publisher, the editors and the reviewers. Any product that may be evaluated in this article, or claim that may be made by its manufacturer, is not guaranteed or endorsed by the publisher.

## Abbreviations

EVD: external ventricular drain
CSF: cerebrospinal fluid
ICH: intracerebral hemorrhage
ICP: intracranial pressure
ICU: intensive care unit
SAH: subarachnoid hemorrhage.
