# Supplementary material for: Risk Factors Promoting External Ventricular Drain Infections in Adult Neurosurgical Patients at the Intensive Care Unit—A Retrospective Study
Source: Front Neurol. 2021 Nov 8;12:734156. doi: 10.3389/fneur.2021.734156 (PMC8631749; doi:10.3389/fneur.2021.734156)
Supplement: Supplementary file 1 [file Table_1.pdf]

Supplementary Table 1 Characteristics of patients with intrathecal vancomycin application with contamination or negative microbiological analysis

Patient	Indication	No-infection subgroup	CSF cell count (per µl)	Lactate (mmol/L)	CSF/Serum Glucose ratio	CRP (mg/dL)	WBC – count (10^9/L)	Temperature (°C)	Systemic infection at the time of vancomycin start
33	SAH	neg. CSF-culture	1600	3.2	0.58	1.9	10.7	38.3	no systemic infection
34	SAH	neg. CSF-culture	1128	7.1	0.16	9.2	13.9	38.5	no systemic infection
35	SAH	neg. CSF-culture	912	3.6	0.40	0.4	12.5	38.7	urinary tract infection
36	SAH	neg. CSF-culture	1971	5.6	0.24	1.6	7.8	38.0	Pneumonia
37	SAH	neg. CSF-culture	308	2.5	0.62	2.1	9.6	38.2	no systemic infection
38	SAH	neg. CSF-culture	2496	6.2	0.40	17.3	6	36.1	Pneumonia
39	SAH	neg. CSF-culture	876	2.5	0.54	7	7.8	37.5	Pneumonia
40	SAH	neg. CSF-culture	4779	5.6	0.44	12.8	6.7	36.5	Urinary tract infection
41	ICH	neg. CSF-culture	668	3.8	0.43	12.6	9.2	38.0	no systemic infection
42	SAH	neg. CSF-culture	456	5.1	0.41	3.8	13.3	37.4	Pneumonia
43	SAH	neg. CSF-culture	859	2.6	0.54	0.1	10.5	38.1	no systemic infection
44	SAH	neg. CSF-culture	2645	4.3	0.40	26.6	13.1	36.2	Urinary tract infection, Pneumonia
45	SAH	contamination	995	2.2	0.58	17.8	9.4	35.7	no systemic infection
46	SAH	contamination	-	-	-	3.8	12.2	37.3	no systemic infection
47	Tumor	contamination	533	5.5	0.34	10.1	12.4	39.3	Pneumonia
48	SAH	contamination	1024	3.0	0.40	0.6	9.2	38.4	Pneumonia
49	SAH	contamination	10	3.4	0.51	0.8	6.8	37.4	no systemic infection
50	SAH	contamination	161	3.3	0.43	1.3	10.4	36.9	no systemic infection
51	Trauma	contamination	18	3.6	0.42	3.5	8.6	37.4	no systemic infection

CRP C-reactive protein, *CSF* cerebrospinal fluid, *ICH* intracerebral hemorrhage, *SAH* subarachnoid hemorrhage, *WBC* white blood cell

Supplementary Table 2 Clinical and laboratory data of patients with EVD associated infection (n=32)

Parameters*	Number of patients (%)
Tmax above or equal 37.5°C	24 (75%)
Cell count above 4 per µl	31 (96.9%)
Protein level above 45mg/dL	16 (50%)
Lactate above 2.1mmol/L	25 (78.1%)
Glucose <50% of systemic glucose	15 (46.9%)
CRP above 3mg/dL	18 (56.2%)

*Patients can be assigned into multiple categories

CRP C-reactive protein, *EVD* external ventricular drainage.

Tmax highest body temperature on the day of infection.
